# Acid stress mediated adaptive divergence in ion channel function during embryogenesis in *Rana arvalis*

**DOI:** 10.1038/srep14201

**Published:** 2015-09-18

**Authors:** Longfei Shu, Anssi Laurila, Katja Räsänen

**Affiliations:** 1 Eawag, Department of Aquatic Ecology, Switzerland and ETH Zurich, Institute of Integrative Biology, Switzerland; 2 Animal Ecology/Department of Ecology and Genetics, Evolutionary Biology Center, Uppsala University, Sweden

## Abstract

Ion channels and pumps are responsible for ion flux in cells, and are key mechanisms mediating cellular function. Many environmental stressors, such as salinity and acidification, are known to severely disrupt ionic balance of organisms thereby challenging fitness of natural populations. Although ion channels can have several vital functions during early life-stages (e.g. embryogenesis), it is currently not known i) how developing embryos maintain proper intracellular conditions when exposed to environmental stress and ii) to what extent environmental stress can drive intra-specific divergence in ion channels. Here we studied the moor frog, *Rana arvalis*, from three divergent populations to investigate the role of different ion channels and pumps for embryonic survival under acid stress (pH 4 *vs* 7.5) and whether populations adapted to contrasting acidities differ in the relative role of different ion channel/pumps. We found that ion channels that mediate Ca^2+^ influx are essential for embryonic survival under acidic pH, and, intriguingly, that populations differ in calcium channel function. Our results suggest that adaptive divergence in embryonic acid stress tolerance of amphibians may in part be mediated by Ca^2+^ balance. We suggest that ion flux may mediate adaptive divergence of natural populations at early life-stages in the face of environmental stress.

Eukaryotic cells are highly compartmentalized, wherein protons and other ions (e.g. Na^+^, K^+^ and Ca^2+^) play a crucial role in signaling, maintaining the structure and function of proteins, and storing energy as an electrochemical gradient across the membrane^1^. Ion channels and pumps, which are responsible for ion flux, have been extensively investigated in relation to nervous systems^2^,^3^. However, relatively little is known on the function of specific channels and pumps during embryogenesis. Studies in model systems, such as mouse, chicken and *Xenopus*, indicate dynamic expression of ion channels and pumps during embryogenesis^4^,^5^,^6^—whereas studies on natural populations facing variable ecological conditions are essentially missing.

This gap is particularly important as embryos of many aquatic taxa develop in direct contact with the external environment and are highly influenced by environmental stressors, such as salinity, temperature and pH^7^,^8^,^9^, which mediate their negative effects via the disruption of ion balance and thereby affect the reproductive success and viability of natural populations. Moreover, environmental stress can have strong ecological and evolutionary consequences on natural populations and cause strong selection at short time scales^10^. It is therefore of key interest to investigate how developing embryos maintain proper intracellular conditions under stressful conditions, and whether environmental stress can cause selection on ion channels and pumps during embryogenesis. Most studies so far have focused on the constitutive role of ion channels and pumps during early embryogenesis, such as neural induction, cavitation or gap junctions^11^. To our knowledge, no studies have investigated the role of environmental stress on ion channels and pumps during embryogenesis. Moreover, very few studies have investigated intra-specific variation (e.g. variation within and among populations) in ion balance in natural populations – in particular in relation to early life stages (see^12^,^13^ in adults).

One potential source of environmental stress, that may impose strong selection on ion channel function, is environmental acidification. Environmental acidification is a major environmental problem in both freshwater and marine ecosystems (e.g.^14^,^15^). The negative effects of acid stress are, in part, mediated via the disrupted ion balance, as shown in fish^16^, larval amphibians^17^,^18^, sea urchins^8^ and marine phytoplankton^19^. However, how acid stress affects functioning of ion channels and pumps during embryogenesis has not been studied to date. This is important because, for instance in fish and amphibians, acid stress typically causes reduced reproductive success due to high embryonic mortality (e.g.^14^,^16^). Amphibians can be strongly negatively affected by acidity at all life-stages (reviewed^14^). The strongly reduced embryonic survival under acidic conditions has traditionally been explained by a “curling defect”, whereby embryos develop, but become tightly curled within the egg coat and, finally, fail to hatch under acidic conditions^20^,^21^. Based on egg coat manipulation experiments, this effect has been suggested to be mediated via chemical alterations in the maternally derived egg coats^20^,^22^. Evidence further indicates that adaptive divergence among and within species arises via egg coat mediated maternal effects^22^,^23^. However, the potential role of disrupted embryonic ion channel function in acidity induced mortality of embryos, and potential adaptive divergence in ion channels and pumps, has not been studied to date.

Here, we combined a common garden laboratory experiment (rearing of embryos at different pHs) with a pharmaceutical approach (ion channel manipulation) to study variation in ion channel and pump function (the general functional role of ion channels) at benign (pH 7.5) and stressful (pH 4) conditions among three populations of moor frog (*Rana arvalis*) known to differ in embryonic acid tolerance^24^. Specifically, we used ion channel blockers to manipulate four ion channels and pumps that are expected to be responsible for maintaining H^+^, Na^+^ and Ca^2+^ balance. We focused on these channels and pumps for the following reasons: H^+^ because pH directly indicates the concentration of H^+^ ions, whereby lower pH reflects higher H^+^ concentration; Na^+^ because acidic pH can inhibit Na^+^ uptake and cause passive Na^+^ loss in fish and amphibians^16^,^17^,^25^, subsequently having sub-lethal and lethal effects and, finally, Ca^2+^ because water hardness (primarily reflecting Ca^2+^ ion concentration) affects acid stress tolerance in fish and amphibians and acidified surface waters are typically soft (low Ca^2+^ concentration^18^,^26^,^27^). We predicted that i) if these ion channels are generally important during embryogenesis, blocking the relevant ion channels should increase mortality of embryos, ii) if the ion channels are important under acidic conditions, these effects should be pH dependent, and iii) if populations adapted to different pH conditions have been exposed to divergent selection on ion channels and pumps, embryos from different populations should show divergent responses to our blocking treatments.

## Results

### Variation in acid stress tolerance and the role of egg jelly

We initially tested embryonic survival in both presence (henceforth, jellied) and absence (henceforth, de-jellied) of the gelatinous egg jelly coats that surround *R. arvalis* embryos, because i) under acidic conditions jelly envelopes typically prevent the embryos from hatching (the curling defect) and jelly removal may either increase or decrease embryonic survival^22^,^23^; ii) jelly envelopes also serve as a protective barrier of embryos from environmental hazards^28^, therefore they could potentially affect our inhibitor treatments.

Overall, acid treatment (pH 4) strongly reduced embryonic survival (compared to the neutral, pH 7.5 treatment), but the populations differed in their pH tolerance: embryos from the population T had over two fold higher survival at pH 4 than embryos from the S population, and B population was intermediate (when jelly was intact; Table S1, Fig. S1). In general, jelly removal had significant effects on hatching success, as indicated by several strong jelly treatment effects (Fig. S1, Table S1). Furthermore, jelly removal removed differences in acid stress tolerance between the S and B population, whereas the T population had higher survival at pH 4 even after jelly removal (Fig. S1). This result indicates that part of the among population differences in embryonic acid stress tolerance at extreme acidic pHs are independent of jelly coats.

There was a significant jelly × inhibitor × population interaction (*P* = 0.025; Table S1). Furthermore, jelly clearly affected effectiveness of the inhibitor treatment in many cases (i.e. hatching success was reduced more strongly in the inhibitor treatments when jelly was removed compared to intact jelly treatment; Fig. S1). As our focus here is on ion channels, we in the following focus mainly on the dejellied treatments.

### Ion channels and pumps

To investigate the potential role of ionic and acid balance at neutral and acidic pH for embryonic survival, we blocked acid-sensing ion channels (ASICs) by a Amiloride (Ami) treatment^29^, Na^+^/K^+^-ATPase (Sodium pumps) by a Ouabain (Oua) treatment^30^, and Ca^2+^ ion channels by Verapamil (Ver, L-type voltage-dependent calcium channels specific inhibitor^31^) and Lanthanum (Lan, a general calcium channel inhibitor^32^) treatments.

**Figure 1:**
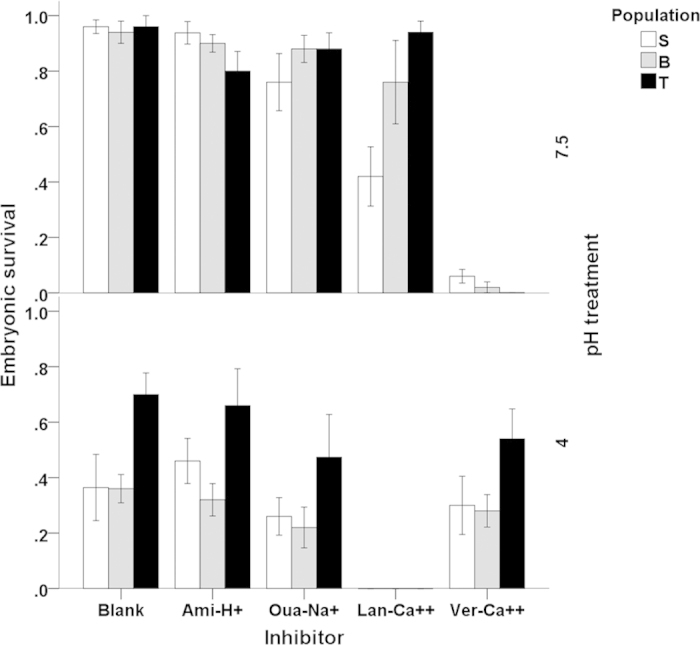
Effects of two pH (7.5: upper panel and 4: lower panel) and five inhibitor (Blank, Ami, Oua, Lan and Ver) treatments on embryonic survival (mean ± SE) for three *R. arvalis* populations: the neutral origin (S), the intermediate pH origin (B) and the acid origin (T) population.

Acidic pH strongly reduced embryonic survival in all populations also in the dejellied treatment but, as indicated by a significant pH × population interaction (Table 1), this effect was much weaker in the acid origin T population (Fig. 1). We found strong Inhibitor main effects as well as significant pH × Inhibitor and Population × Inhibitor interactions (Table 1). It was apparent that Ver treatment had strong negative effects at pH 7.5 in all populations, whereas the Lan treatment had strong negative effects at pH 4 in all populations (Fig. 1). To establish the nature of the pH interactions, we next investigated the potential role of the inhibitors in embryonic performance within each pH treatment separately (Tables 2 and 3).

Within pH 7.5 (Table 2), there was a highly significant Inhibitor main effect and Inhibitor × Population interaction, indicating that differences among the inhibitor treatments as well as among population divergence in inhibitor function was expressed at neutral pH. In contrast, at pH 4 Population main effect was significant, but there was no significant Inhibitor main effect or Inhibitor × Population interaction (Table 2).

Taken together, these results indicate that the T population had consistently higher acid tolerance, but that in terms of inhibitor effects, the populations responded similarly to acidic pH (Fig. 1). Moreover, it is clear that inhibitor effects are strongly pH dependent. To gain insight to the nature of the interactive effects, we next compared the impacts of the different inhibitor effects by Population and pH treatment (Table 3; Fig. 1)

### General calcium flux mediating channels

The Lan treatment had different effects under different pHs (Fig. 1, Table 2). Strikingly, at pH 4, in all populations almost all embryos in the Lan treatment failed to hatch (Fig. 1; Table 3) and most of the embryos died before day 7 (Shu, pers. obs). At pH 7, populations differed in sensitivity to the Lan treatment: embryonic survival was reduced by the Lan treatment only in the neutral origin S population, while T and B populations were not affected (Fig. 1; Table 3).

These results indicate that Ca^2+^ influx is crucial for embryonic acid stress tolerance and suggest among population divergence in calcium channel function in *R. arvalis*. However, as Lanthanum chloride is a general calcium channel inhibitor, we could not specify which type(s) of calcium channels may have been under selection by acidic stress and contributed to embryonic fitness.

### Acid-sensing ion channels (ASICs)

In general, we found no significant effects of the Ami treatment (compared to the blank) on survival at either of the pH treatments (Fig.1, Table 3). ASICs are generally considered to be activated under acidic pH by extracellular protons and play crucial role in acid sensing^33^, but our data suggests that ASICs did not play an essential role during early embryonic development when facing acidic stress in *R. arvalis*.

### Na^**+**^/K^**+**^-ATPase (Sodium pumps)

There was a slight reduction in survival in the Oua treatments (Fig. 1). Although there was no significant Population × Inhibitor interaction at pH 4 (Table 2), the pairwise comparisons indicated that the Oua treatment reduced survival (though marginally) only in the T population at pH 4 (Fig. 1, Table 3). This subtle divergence indicates possible divergence in sodium pump function in response to acidic stress, although its contribution to embryonic fitness seems relatively weak at this point.

Intriguingly, however, all larvae (in all populations and both pH treatments) in the Oua treatment died at Gosner stage 22/23 (the stage prior to gill absorption; Gosner 1960; Shu pers. obs., data not shown). This indicates that Na^+^ balance becomes important at early larval stages in amphibians, in accordance with previous studies in fishes^16^,^25^.

### L-type voltage-dependent calcium channels

In contrast to the Lan treatment, the Ver treatment dramatically reduced hatching at pH 7.5, but had no significant effect at pH 4, and this effect was seen in all three populations (Fig. 1, Table 3). This indicated that L-type voltage-dependent calcium channels are not involved in embryonic acid stress tolerance, but have a crucial function during embryonic development at neutral pH. In addition, the fact that the Ver treatment did not have any significant effects on embryonic fitness at pH 4 suggests that this channel may be naturally blocked when challenged by acidic stress. Previous studies on *X. laevis* suggested that L-type calcium channels play a role in neural induction of amphibian embryos^34^. Therefore, it would be interesting to further investigate that if L-type calcium channels indeed can be blocked by acidic stress, how could the embryos still successfully develop without the aid of this channel.

## Discussion

We found that Na^+^ and, in particular, Ca^+^ ion channels and pumps play diverse roles during early embryogenesis and embryonic responses to acid stress. Intriguingly, our results further show among population differences in Ca^+^ ion channel function (in particular, effect of Lan treatment on S population at pH 7.5), indicating that environmental acidification may drive adaptive divergence of ion channel function at early life-stages. We show, for the first time, that embryonic acid stress tolerance in amphibians is dependent on Ca^2+^ ion flux and that different calcium channels are activated under different pH conditions.

### Ca^**2+**^ influx is essential for embryonic survival under acidic pH

Maintaining an optimal intracellular pH is crucial for homeostasis of organisms, but this can be affected by various environmental conditions, such as salinity and environmental acidity^1^. Although it is not clear to date how environmental stress affects functioning of ion channels and pumps during embryogenesis, current evidence from various systems^8^,^16^,^17^,^19^ suggests that the negative effects of acid stress are in part mediated via the disrupted ion balance of H^+^ and Na^+^.

Therefore, a reasonable assumption would be that H^+^ and Na^+^ channels are important also for embryonic acid stress tolerance in amphibians and other taxa directly exposed to environmental pH. However, we found no support for the above hypothesis. Instead, we showed that embryonic acid stress tolerance in *R. arvalis* was Ca^2+^ flux dependent: blocking of Lanthanum chloride sensitive calcium channels caused almost 100% mortality under acidic conditions, whereas Ca^2+^ flux seemed to be less essential under neutral conditions (Fig. 1). This suggests that under acid stress, embryos need to uptake Ca^2+^ from the environment to maintain ionic balance and stay alive.

Our findings indicate that Ca^2+^ in acidic environments should reduce the negative effects of acid stress. This is in accordance with previous experimental studies that manipulated calcium content in experimental settings, as well as the (sometimes) positive consequences of liming that have been used to counteract acidification. For example, in brown trout *Salmo trutta* increased external concentrations of calcium increased egg survival from 10% to 90%^35^. Increased calcium levels have been found to increase survival at acidic pH under laboratory conditions also in several amphibians^17^,^18^. Likewise, liming of acidified ponds increased embryonic survival of *R. arvalis* and common frog *R. temporaria*^36^,^37^.

Taken together, our results show, to our knowledge for the first time, that Ca^2+^ influx is essential for embryonic survival under acidic pH. Given that Ca^2+^ has a dual role in both signaling and ion balance during embryogenesis^38^, we suggest that the role of Ca^2+^ dynamics during embryogenesis should be considered more comprehensively, in particular in the context of environmental stress, such as acidity.

### New insight to components of embryonic acid stress tolerance

Prior work in amphibians identified the “curling defect” as the main mechanism drastically reducing embryonic survival at acidic pH^14^,^20^,^21^. Moreover, studies on both between and within species in embryonic acid stress tolerance suggested that adaptive divergence is driven primarily by egg coat mediated maternal effects^22^,^23^. The present study adds a new aspect to understanding adaptive responses to environmental acidification and suggests that the ion channels influencing embryonic performance may also be under divergent natural selection by environmental pH.

In particular, we found that Lanthanum chloride sensitive Ca^2+^ channels influenced embryonic acid stress tolerance. This indicates that maintaining a certain level of intracellular Ca^2+^ concentration is crucial for embryonic fitness under acidic stress. Moreover, we found a clear pattern of population divergence at neutral conditions (Fig. 1, Table 1): embryos from the acid origin (T) and the intermediate pH origin (B) population were not affected by the Lan treatment at pH 7.5, but the embryos from the neutral origin (S) population had strongly reduced survival in this treatment combination (Fig. 1, Tables 1 and 3). This suggests a significant functional divergence of Ca^2+^ channels across populations. However, adaptive divergence in Lan treatment was not manifested at pH 4. This may be because (for logistic reasons) only one—relatively high—concentration of the ion channel blocker was used and adaptive divergence may have been evident at lower concentrations of Lanthanum chloride.

**Figure 2:**
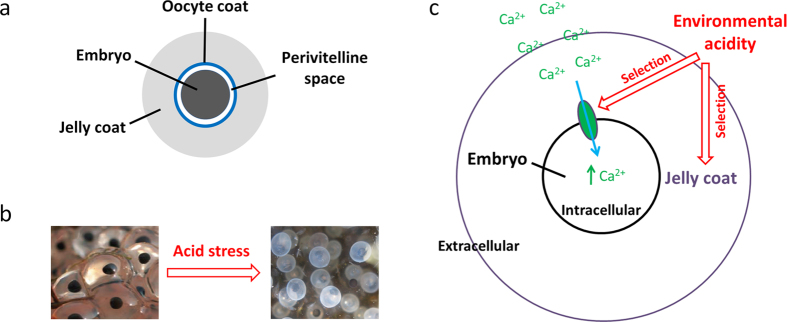
Key components of embryonic acid stress tolerance in amphibians. (**A**) A schematic presentation of an amphibian embryo. Embryos are surrounded by a perivitelline space, and the egg coats, which can be divided into the innermost oocyte coats (called fertilization envelopes, FE, after fertilization) and the outer gelatinous coats (jelly coats); (**B**) Visualization of effects of environmental acidity on *R. arvalis* embryos, with healthy embryos at the left and embryos dying under acid stress on the right; (**C**) A schematic presentation of the main mechanisms for pH mediate selection on embryonic acid stress tolerance in amphibians (see main text). Photos of *R. arvalis* copyright Katja Räsänen.

Nevertheless, taken together with the evidence for divergence via egg coat mediated adaptive maternal effects^22^,^23^, we propose that environmental acidity can simultaneously drive adaptive divergence in both egg coats and embryonic ion channels—in particular Ca^2+^ channels (Fig. 2). First, in order to develop normally in acidic environments, embryos have to be able to maintain intracellular ionic and acid balance, which imposes selection on Ca^2+^ channel function (Fig. 2). Second, as the jelly coats are strongly negatively affected by acidic pH, trapping embryos inside the jelly and resulting in failure to hatch, there is selection on the egg coats—possibly in their composition^39^,^40^. Therefore, we propose that at extreme acidic conditions—such as those the T population is exposed to—there is simultaneous selection on Ca^2+^ channel and egg coats. Explicit tests of this hypothesis are needed and would shed light on how selection simultaneously acts on offspring traits and maternal effects.

## Materials and Methods

### Study system

*R. arvalis* is a widely distributed anuran in the western Palearctic and inhabits a wide range of pH’s^41^. The species breeds in early spring, after snow melt, and females produce a single clutch of eggs per year, laid directly in water. The clutch size ranges from about 500–2000 eggs in our study region^14^. Embryos are surrounded by maternally derived egg coats, which can be divided into the fertilization envelope (FE) and the gelatinous outer layers (so called Jelly coats) (Fig. 2a). The species can inhabit a wide range of pHs (from 4 to 9)^14^ and has become a model system for studies on adaptation to acidification^23^,^24^,^39^,^40^.

Three populations breeding in permanent ponds in southwestern Sweden, and known to vary in embryonic acid tolerance, were used in this study (Figure S1, details are provided in^24^). The pH in these ponds ranged from 4.0 in the most acid tolerant population (Tottajärn, T, 57°60N, 12°60E) to 6.1 in an intermediately tolerant (Bergsjö, B, 58°20N, 13°48E) to 7.3 in a highly acid-sensitive population (Stubberud, S, 58°46N, 13°76E). Site T is situated centrally within an area that has been heavily affected by anthropogenic acidification since the early 1900s^42^, whereas site S has remained unaffected by acid rain due to limestone bedrock^42^. Site B was heavily acidified until 1987 (lowest pH measured pH 4.2), but is since being limed regularly. It has a somewhat fluctuating pH around pH 6 (Annica KarLsson, pers. comm., Västra Götaland county board).

In each population, five freshly fertilized clutches were collected in the breeding ponds within ca. 30 min of egg laying (i.e. prior to first cell division and when eggs or egg coats have not yet absorbed substantial amounts of water). Each site was continuously checked during the sampling night. Only freshly laid clutches (within 30 min of egg laying) were collected and immediately transferred to reconstituted soft water (RSW, pH 7.2–7.613). The embryos were maintained cool to slow down embryonic development, and transported to the laboratory at Uppsala University within one day from collection.

The methods were performed in accordance with the guidelines and regulations of Uppsala University. All experimental protocols were approved by the Västra Götaland county board and the Ethical committee for animal experiments in Uppsala County.

### Experimental procedures

#### Jelly removal

Jelly can serve as a protective barrier to environmental hazards^28^,^43^,^44^ and therefore could also potentially influence successful application of the inhibitors which consist of relatively large molecules. Therefore, half (i.e. 100 eggs) of the eggs in each clutch were de-jellied manually using watchmaker’s forceps^45^,^46^. Each egg was inspected under a stereomicroscope to assure that the jelly removal left the FE apparently intact. Any damaged or unfertilized eggs were disregarded prior to the experiment. This resulted in a total of 1, 500 de-jellied eggs.

#### Inhibitor preparation

Amiloride hydrochloride (Amiloride hydrochloride hydrate 95%, 2016-88-8), Ouabain octahydrate (Ouabain octahydrate 95% 11018-89-6), Lanthanum chloride (Lanthanum(III) chloride anhydrous, beads, −10 mesh, 99.9% trace metals basis, 10099-58-8) and Verapamil hydrochloride (Verapamil hydrochloride ≥99% (titration), powder, 152-11-4), purchased from Sigma-Aldrich Co. LLC. Sweden, were used as ion channel blockers. Deionized distilled water (Milli Q water purification system, Millipore) was used for preparation of stock solutions (10 mM) and experimental solutions. For logistic reasons, only one concentration (0.1 mM) was used within each inhibitor treatment. This concentration was chosen based on those commonly used in the literature in ion channel studies^29^,^30^,^31^,^32^. Inhibitor stock solutions were prepared fresh, immediately prior to the experiment. 160 eggs from each family were used for each inhibitor treatment, resulting in a total of 3 000 eggs for the following experiment.

#### Rearing of embryos

The acid tolerance test of each clutch within the three populations were performed in a walk-in climate room (~16 °C) with 17 L: 7D photoperiod and embryos were reared at two pH treatments (acid: pH 4.0, neutral: pH 7.5). Reconstituted soft water (RSW) was used as the experimental medium, similar to our former work^23^,^24^. The pH in the neutral treatment was not adjusted (nominal pH of RSW is 7.2–7.6 when organisms are in the water), whereas in the acid treatment it was adjusted with 1M H_2_SO_4_ in 200 L containers at least two days prior to use. Embryos were placed in the experimental treatments within three hours after arrival in the laboratory. Embryos were reared singly in PP plastic vials (0.25 L), containing 0.1 L of treatment water. No water change was conducted during the experiment. Embryos were reared from fertilization to day 12 (when all surviving embryos should have hatched).

#### Experimental setup

The whole experiment was performed as a factorial 2 × 3 × 5 × 5 × 2 nested randomized design, with two pH treatments (pH 4.0, and 7.5), three populations (T, B and S), five clutches (i.e. full-sib families) per population, five inhibitor treatments (Blank control, Amiloride, Ouabain, Lanthanum chloride and Verapamil) and two jelly treatments (jellied and dejellied). Each family treatment combination was replicated ten times, resulting in a total of 3, 000 experimental units. The replicates were fully randomized over the experiment shelves. Any unfertilized eggs (i.e. if no cell division was apparent eggs were assumed to be unfertilized) were determined at day 3 and excluded from the analyses of hatching success. However, fertilization success was very high (near 100%). Hatching was recorded visually at each day, but only final survival (day 12) was used in the statistical analyses.

#### Statistical analyses

To reduce model complexity, the data was grouped by family for statistical analyses. Hence, family (instead of individual vials) was the unit of analysis (resulting in five replicates/population treatment combination). The response variable was survival to day 12 (hatched/total embryos). The data was analyzed with generalized linear effects models (GLM) with binomial error structure and logit link function with the GENMOD procedure in SAS 9.3 (SAS Institute, Inc.). The full model (across the whole experiment) included jelly, pH treatment, population, inhibitors and all possible interactions as fixed factors. However, as the jelly clearly affected the efficiency of the inhibitor treatments (Fig. S1), the analyses testing the effects of inhibitors were conducted within the dejellied treatment.

The submodels within the dejellied treatment included pH treatment, population, inhibitors and all possible interactions as fixed factors. Some modifications had to be made due to near complete mortality in the Lan treatment at pH 4 for all populations, and hence complete separation of treatment responses. The models including this treatment combination failed to converge. Hence, this treatment was excluded in some of the analyses and only subsets of data were analyzed. For comparative interpretation of the data in the Lan-pH 4 treatment, we use visual interpretation (see Fig. 1, Figure S1). Three main sets of data analyses were conducted: 1) a full model including jelly treatments, but excluding the Lan treatments (Table S1), 2) a model within the dejellied treatment, excluding the Lan treatments (Table 1) and 3) models within each pH treatment, whereby also Lan was included in the pH 7.5 treatment, but excluded in the pH 4 treatment (Table 2). The effects of inhibitors were rested using planned contrasts on LSmeans (control vs. a given inhibitor) with a Dunnett’s test for each population within a given pH treatment (Table 3).

## Additional Information

**How to cite this article**: Shu, L. *et al*. Acid stress mediated adaptive divergence in ion channel function during embryogenesis in *Rana arvalis*. *Sci. Rep*. **5**, 14201; doi: 10.1038/srep14201 (2015).

## Supplementary Material

Supplementary Information

## Figures and Tables

**Table 1:** Generalized linear model of embryonic survival in response to inhibitor treatments.

*Fixed effects*	*df*	*χ*^2^	*P*
Population	2	0.65	0.723
pH	1	0.00	0.999
Inhibitor	3	282.27	**<0.001**
pH × Population	2	13.96	**<0.001**
pH × Inhibitor	3	193.78	**<0.001**
Population × Inhibitor	6	14.00	**0.030**
pH × Population × Inhibitor	6	10.52	0.104

Results are shown for three *R. arvalis* populations under two pH (pH 4 and pH 7.5) and the four inhibitor (Blank control and Ami, Oua and Ver) treatments. The treatment Lan had was excluded from the model because the model did not converge due to complete mortality at pH 7.5. Significant effects are highlighted in bold.

**Table 2:** Generalized linear model of embryonic survival by pH treatment.

*Fixed effects*	A) pH 7.5 (all inhibitors)			B) pH 4 (without Lan)		
	*df*	*****χ*^2^	*P*	*df*	*χ*^2^	*P*
Population	2	0.34	0.842	2	14.55	**<0.001**
Inhibitor	4	260.00	**<0.001**	3	2.77	0.428
Population × Inhibitor	8	24.16	**0.002**	6	0.58	0.748

At pH 7.5, results are shown for three *R. arvalis* populations under all five inhibitor treatments. At pH 4, the complete mortality of embryos in the Lan treatment resulted in problems with model convergence and Lan was, hence, excluded from the pH 4 analysis. Significant effects are highlighted in bold.

**Table 3:** Dunnett’s tests of pairwise differences in least square means from a generalized linear model of survival of *R. arvalis* embryos between a given inhibitor treatment and the control (blank) for each population within each pH.

*Contrast*	*Population*	A) pH 7.5		B) pH 4	
		*z*	*Adj P*	*z*	*Adj P*
Amiloride vs. Blank	S	−0.01	1.000	0.82	0.750
	B	−0.26	0.995	−0.42	0.955
	T	−1.59	0.270	−0.77	0.764
Ouabain vs. Blank	S	−1.55	0.289	−1.06	0.586
	B	−1.53	0.314	−1.53	0.293
	T	−1.15	0.535	−2.426	*0.060*
Verapamil vs. Blank	S	**−6.38**	**<0.001**	−0.37	0.966
	B	**−5.71**	**<0.001**	−0.86	0.730
	T	**−6.56**	**<0.001**	−0.95	0.641
Lanthanum vs. Blank	S	**−4.09**	**<0.001**	n.a. (due to complete mortality in Lan at pH 4 in all populations)	
	B	−1.53	0.310		
	T	−0.46	0.914		

The inhibitor effect tests exclude the Lan treatment, which showed near complete mortality in all populations.
