# Supplementary material for: Acid stress mediated adaptive divergence in ion channel function during embryogenesis in Rana arvalis
Source: Sci Rep. 2015 Sep 18;5:14201. doi: 10.1038/srep14201 (PMC4585641; doi:10.1038/srep14201)

Supplementary Information

Acid stress mediated adaptive divergence in ion channel function during embryogenesis in *Rana arvalis*

Longfei Shu[1*], Anssi Laurila[2] and Katja Räsänen[1]

Affiliations:

[1]Eawag, Department of Aquatic Ecology, Switzerland and ETH Zurich, Institute of Integrative Biology, Switzerland

[2]Animal Ecology/Department of Ecology and Genetics, Evolutionary Biology Center, Uppsala University, Sweden

Corresponding author:

Longfei Shu

Eawag, Department of Aquatic Ecology, Switzerland and ETH Zürich, Institute of Integrative Biology, CH-8600 Dübendorf, Switzerland

Email: longfei.shu@wustl.edu

***Current address:**

Department of Biology, Washington University in St. Louis, St. Louis, MO 63130, USA

SI Table and figures

SI Table 1. Generalized linear models of embryonic survival in three *R. arvalis* populations in response to four inhibitor (Blank control, Ami, Oua and Ver), two jelly (jelly intact, de-jellied) and two pH (pH 4.0 and pH 7.5) treatments. As the complete mortality of embryos in the Lan treatments at pH 7.5 resulted in lack of convergence of the model, model was ran without the Lan treatment. Significant effects (P < 0.05) are highlighted in bold.

Fixed effect	*df*	χ^2	*P*
pH treatment	1	0.00	1.000
Jelly treatment	1	44.09	**<0.001**
Inhibitor treatment	3	357.11	**<0.001**
Population	2	1.78	0.410
pH × Jelly	1	1.90	0.168
pH × Inhibitor	3	6.45	**<0.001**
Jelly × Inhibitor	3	1.81	0.612
pH × Population	2	18.64	**<0.001**
Jelly × Population	2	8.03	**0.018**
Inhibitor × Population	6	6.45	0.375
pH × Jelly × Inhibitor	3	0.29	0.962
pH × Jelly × Population	2	2.10	0.350
pH × Inhibitor × Population	6	10.01	0.124
Jelly × Inhibitor × Population	6	14.44	**0.025**
pH × Jelly × Inhibitor ×	6	9.10	0.168

SI Figure 1. Survival (mean ± SE) of embryos at two jelly (jelly intact, de-jellied), five inhibitor (Blank control, Ami, Lan, Oua and Ver) and two pH (pH 4.0 and pH 7.5) treatments in three *R. arvalis* populations (S, B and T).

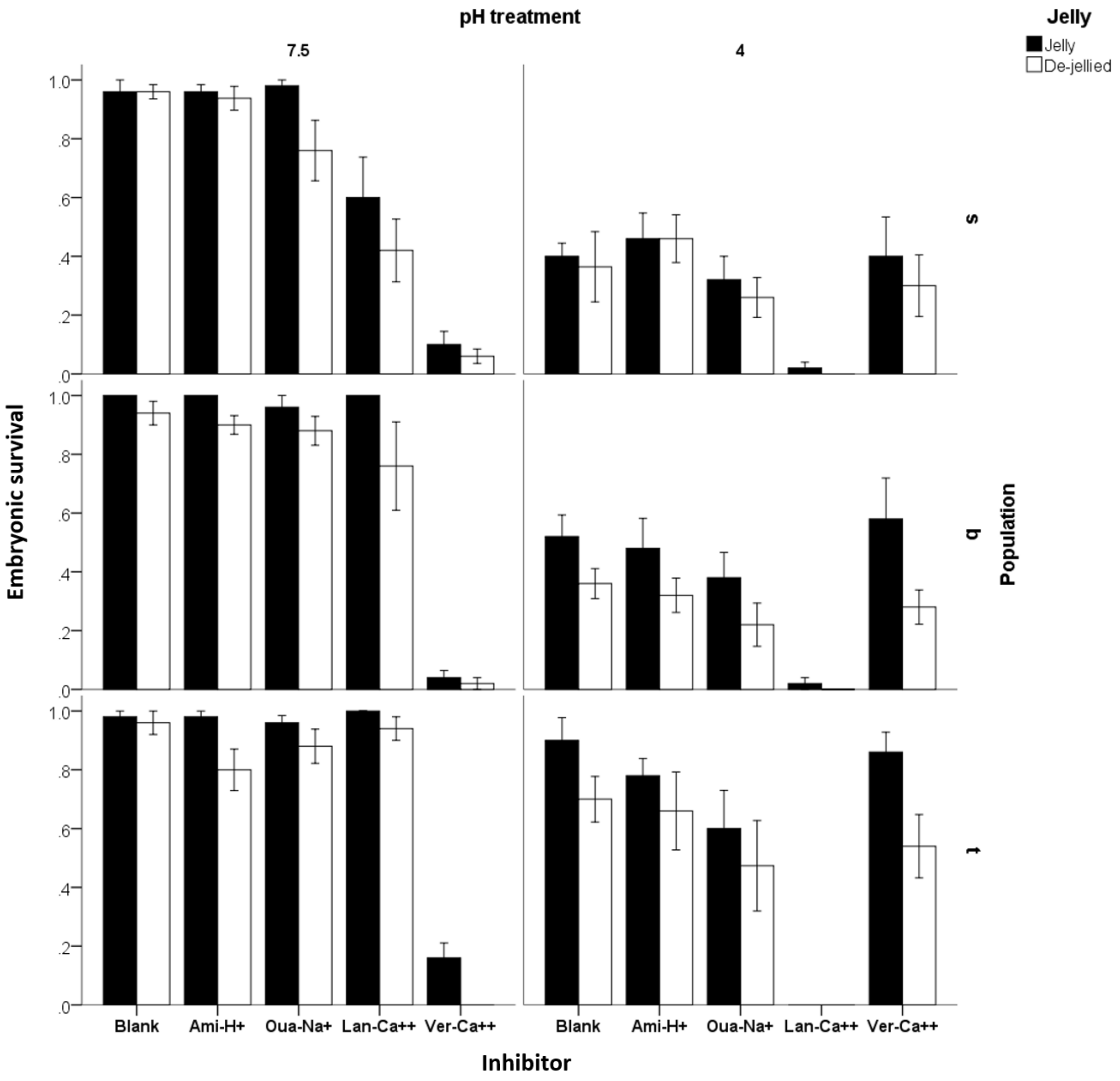

Supplement: Supplementary Information [file srep14201-s1.pdf]
